# Editorial: Sex differences in the brain

**DOI:** 10.3389/fnana.2022.1000121

**Published:** 2022-08-10

**Authors:** Luis Miguel Garcia-Segura, Javier DeFelipe

**Affiliations:** ^1^Cajal Institute, Spanish National Research Council (CSIC), Madrid, Spain; ^2^Cajal Laboratory of Cortical Circuits, Centre for Biomedical Technology (CTB), Madrid, Spain

**Keywords:** sex determination, synapses, estradiol, testosterone, hypothalamus, prefrontal cortex

The difference between the brain of males and females is a matter of general interest from many different perspectives—and one that has been the focus of debate for centuries. Understanding the effects that sex differences can have in the brain will help us better understand how the brain works and provide a better approach to diseases in which prevalence differs between males and females. These differences are thought to be a consequence of not only the influence of sex hormones on multiple regions of the brain during development, but also the direct action of genetic factors present in sex chromosomes (nature), epigenetic modifications in the neural genome, and environmental conditions (nurture). For example, sex hormones have many effects throughout the brain that impact cognitive function, neuroprotection, addiction and blood pressure, among many other features. However, the dimorphic view of the brain—categorizing into female and male brain— is often considered an exaggeration.

The articles in this Research Topic focus on the genetic, hormonal and environmental factors that give rise to sex differences in the organization of the nervous system. The role of master sex determination genes in the generation of sex differences in the nervous system has been mainly studied in invertebrate models. This is the case of the TRA-1 gene in *Caenorhabditis elegans*. In this nematode, there are two male-specific neurons. Furthermore, about 30% of synaptic connections of the neurons shared by both sexes present sexually dimorphic phenotypes characterized by differences in (i) the branching of neural processes, (ii) the pattern of axonal projections and (iii) synaptic function. As reviewed by Kim and Kim, sex differences in the synapses of sex-shared neurons are in most cases mediated by the transcription factor TRA-1, a master regulator gene involved in the regulation of other transcription factors. The expression of TRA-1 in sex-shared neurons depends on the sex of the animal, but is also modulated by other factors, such as age and environmental conditions.

Among the genes regulated by TRA-1 in the nervous system of *C. elegans* are a group of double sex/MAB-3 domain transcription factors (*Dmrt*). *Dmrt* genes are known to be involved in gonadal sex differentiation in a variety of animal species, including nematodes, insects, fish, amphibians, birds and mammals. In the gonads, *Dmrt* genes act as pioneer transcription factors, allowing the action of other transcription factors involved in gonadal differentiation. In somatic cells, *Dmrt* genes integrate information on sex, space and time to determine male or female phenotypes. As reviewed by Casado-Navarro and Serrano-Saiz, who mainly focused on data obtained in *C. elegans* and *Drosophila melanogaster, Dmrt* genes show sex differences in expression in the nervous system, where they are involved in the regulation of neurogenesis, cell death, synaptic pruning and neurotransmitter gene expression, all of which lead to sex differences in the organization of neuronal circuits.

Much less is known about the role of *Dmrt* genes in the sexual differentiation of the mammalian brain. By contrast, there is an abundance of literature on the sex-specific organizational effects of gonadal hormones during critical developmental periods. One of the intriguing unknowns is how these hormonal actions during the development are transformed into permanent sex differences in the adult. In this regard, Lagunas et al. investigated the organizational effects of estrogens and androgens in the pituitary and adrenal glands of male and female rats. They examined the effects of the early postnatal administration of an antagonist (flutamide) of androgen receptors, an inhibitor (finasteride) of 5α-reductase, the enzyme that converts testosterone into its active metabolite dihydrotestosterone or an inhibitor (letrozole) of the enzyme aromatase, which converts testosterone into estradiol. Then, they studied the expression of androgen (AR) and estrogen (ER) receptors in the pituitary and adrenal glands of adult animals. The results show the existence of basal sex differences in the expression of AR and ER in the pituitary and adrenal glands that were modified by early postnatal interference with androgen and estrogen signaling. These findings suggest that neonatal androgen and estrogen signaling exerts long-term effects on the expression of ARs and ERs in adult tissues involved in neuroendocrine regulation, thus determining a sex-specific response of these adult tissues to adult levels of gonadal hormones.

It is not only in invertebrate models that environmental factors affect the sexual differentiation of the nervous system; this also occurs in mammals. As reported by Blanco et al., a low-protein and low-calorie diet during the prenatal period increased the number of neurons in the hypothalamic arcuate nucleus of newborn male rats but did not affect this parameter in newborn females. These findings indicate that environmental factors, such as nutrient availability, affect the generation of sex differences in the mammalian brain. Since the arcuate nucleus, among other functions, contains neuronal circuits that regulate food intake and energy expenditure, it is possible that the observed changes represent an organizational plastic adaptation of these neuronal circuits to environmental food conditions. However, the reason why male animals are more sensitive to these environmental conditions remains to be determined and the same can be said of the molecular mechanisms involved in the interaction between prenatal food availability and the hormonal and sex chromosome genetic program involved in mammalian brain sexual differentiation.

Nutritional conditions during adult life may also have sex-specific effects in the brain. Freire-Regatillo et al. studied the metabolic and hypothalamic response to long-term high fat diet (HFD) in a transgenic Alzheimer's disease mouse model. Transgenic males showed higher levels of murine amyloid β and a greater microglial response to HFD in the hypothalamus compared with wild type and transgenic females. In addition, HFD caused changes in the hypothalamic expression of metabolic neuropeptides that were also different depending on sex and genotype. This suggests that nutritional conditions during adult life may have a sex-specific impact on the metabolic regulation exerted by the hypothalamus under basal and neurodegenerative conditions.

The study of sex differences in the human brain is important if we are to understand the different prevalence in men and women of numerous neurological and psychiatric diseases. However, although sex differences in the volume of some regions of the human brain have been reported, their functional impact in terms of human behavior and cognition is still a matter of debate. In this regard, the analysis of sex differences in cognitive regions of the brain, such as the prefrontal cortex, are of particular relevance. Bruno et al. performed a cytoarchitectonic study of the human dorsolateral prefrontal cortex and identified four new distinct cortical regions located in the anterior superior frontal sulcus and in the middle frontal gyrus. Although two of the newly identified regions occupied larger volumes in women than in men, no sex differences were detected in their basic cytoarchitectonic organization, suggesting a similar functional organization in both sexes despite size differences.

Obviously, the articles collected in this Research Topic represent only a few examples of the many lines of research that are ongoing on sex differences in the brain. It is true that some of the differences reported here, as well as in several other publications, seem to be minor and are thought to have little impact on brain function in health and disease. However, other studies strongly suggest that sex-based differences represent a major factor. Although it may appear a platitude, perhaps it is prudent to conclude that differences may or may not be found depending on which aspects of the nervous system are examined. This clearly suggests that new and more detailed studies on the similarities and differences are needed to provide a better understanding of the brain in general and to explain the sex differences observed in the manifestation of numerous brain diseases.

## Author contributions

Both authors listed have made a substantial, direct, and intellectual contribution to the work and approved it for publication.

## Conflict of interest

The authors declare that the research was conducted in the absence of any commercial or financial relationships that could be construed as a potential conflict of interest.

## Publisher's note

All claims expressed in this article are solely those of the authors and do not necessarily represent those of their affiliated organizations, or those of the publisher, the editors and the reviewers. Any product that may be evaluated in this article, or claim that may be made by its manufacturer, is not guaranteed or endorsed by the publisher.

